# An artificial intelligence-aided scoping review of medicinal plant research in the Fertile Crescent

**DOI:** 10.3389/fphar.2025.1542709

**Published:** 2025-06-03

**Authors:** Rawan Nihad Al-Sammarraie, Hadi Al Mubasher, Mariette Awad, Sally Naalbandian, Nadine Darwiche, Rami Zurayk, Maya Romani, Salma N. Talhouk

**Affiliations:** ^1^ Landscape Design and Ecosystem Management, School of Architecture and Design, Maroun Semaan Faculty of Engineering and Architecture, American University of Beirut, Beirut, Lebanon; ^2^ Department of Electrical and Computer Engineering, School of Architecture and Design, Maroun Semaan Faculty of Engineering and Architecture, American University of Beirut, Beirut, Lebanon; ^3^ University Libraries, American University of Beirut, Beirut, Lebanon; ^4^ Department of Biochemistry and Molecular Genetics, Faculty of Medicine, American University of Beirut, Beirut, Lebanon; ^5^ Department of Family Medicine, Faculty of Medicine, American University of Beirut, Beirut, Lebanon

**Keywords:** medicinal plants, Arab medicine, Islamic medicine, Fertile Crescent, pharmacology, artificial intelligence, herbal medicine

## Abstract

Traditional Arabic and Islamic Medicine (TAIM) originated in the seventh century, but unlike Chinese and Ayurvedic knowledge, TAIM has not evolved through evidence-based research and commercialization. Today, while global interest in traditional medicine is growing, TAIM ancestral knowledge remains unknown and unexplored. The purpose of this study is to provide baseline information on the status of TAIM research to guide future research and contribute to the growth of the sector. The focus of the study is the Fertile Crescent, a region of the Arab World endowed with a rich and diverse eco-geography. The method adopted was a scoping review following the Preferred Reporting Items for Systematic Review and Meta-Analysis (PRISMA) guidelines. The databases used included the Arab World Research Source: Al Masdar, CAB Direct, Iraqi Academic Scientific Journals, MEDLINE, Scopus, Web of Science and Google Scholar. The timeline of the search spanned from the database inception date to June 2024. The search led to 10,171 records which were subsequently reduced to 1,990 publications after deleting duplicates and performing a two-stage screening. Artificial intelligence (AI) technology was used to analyze the data focusing on reported plant species, treatment applications, study types and countries. The Generative Pretrained Transformer 4 (GPT-4) Turbo, a large language model, was used to extract the key features and the results were validated by the researchers. The findings revealed that the types of studies were mostly laboratory-based (86%), while few studies (14%) were field based. The top five treatment applications include cancer (29%), bacterial infections (22%), inflammation (12%), fungal infections (9%), and diabetes (8%). The most notable plant species that were under investigation in the various studies were *Nigella sativa* L. (Ranunculaceae), *Rosmarinus officinalis* L. (Lamiaceae), *Salvia fruticosa* Mill (Lamiaceae), *Teucrium polium* L. (Lamiaceae), and *Thymus vulgaris* L. (Lamiaceae). In this review we discuss our findings which suggest potential avenues for further developing TAIM research and exploring the development of botanical drugs. Our findings also revealed that the number of ethnobotanical studies was limited suggesting an urgent need to prevent the loss of ancestral knowledge by formalizing it through evidence-based research and policy guidelines. Addressing these gaps through interdisciplinary collaboration and improved data-sharing mechanisms will be crucial for advancing TAIM research and medicinal plants.

## 1 Introduction

Traditional Arabic and Islamic Medicine (TAIM) is unique in that Islamic scholars in the seventh century integrated Graeco-Roman, Chinese, Persian, and Ayurvedic medical knowledge with Arab knowledge (Alrawi and Fetters, 2012). However, today while TAIM remains informal and fragmented, traditional plant-based medicine originating in other parts of the world are widely investigated, commercialized and practiced, especially in rural areas and developing economies (Azaizeh et al., 2006; Manaf et al., 2017). The need to strengthen and formalize TAIM stems from the fact that traditional and complementary medicine plays a significant role in healthcare in the Arab World, with a projected market growth of 23% annually from 2020 to 2027 (Grand View Research, 2023). Yet, TAIM research does not seem to be strengthening the sector. Several reasons may explain the stagnation of the sector, these probably include poor collaboration between traditional healers, ethnobotanists, pharmacists, and biomedical researchers as well as limited access to standardized, consolidated databases on medicinal plant usage in the region (Azaizeh et al., 2006). To advance the TAIM sector, collaboration, data sharing, and robust, evidence-based research on the identity, safety, toxicity, and dosage of used medicinal plants are required (AlRawi et al., 2017; Chaachouay and Zidane, 2024; Naja et al., 2015; Zbeeb et al., 2024). Focusing on the Fertile Crescent region of the Arab World, this review seeks to fill a gap by presenting a comprehensive overview of TAIM research.

The Fertile Crescent is a region of the Arab World which today includes Iraq, Syria, Lebanon, Jordan, and Palestine. It strategically bridges Asia, Africa, and Europe and has played a pivotal role in the development of ancient civilizations due to its fertile soil, access to water sources, and proximity to trade routes connecting these continents. In fact, the Fertile Crescent is often referred to as the “cradle of civilization” because of its ancient and rich cultural and historical heritage (Lawrence et al., 2016). Ecologically, the Fertile Crescent presents a unique combination of fertile plains, mountains, and deserts (Hekmat and Al-Obeidi, 2019) that have led to a diverse range of climates and ecosystems that support a wide range of flora and fauna. Agriculturally, the Fertile Crescent’s fertile soil and favorable climate have made it ideal for the cultivation of crops, including medicinal plants, which have been used for centuries by ancient civilizations to treat various ailments and diseases (Zeder, 2024). The identification and analysis of 1,990 studies spanning nearly a century (1933–2024) in this review offers the largest compiled dataset on medicinal plants research in the Fertile Crescent. To ensure a timely publication of the studies collected, we resorted to AI-based text analysis, specifically GPT-4 Turbo, because manual data extraction is time-consuming and inconsistent. AI technology enables rapid processing of large datasets, ensuring a comprehensive and standardized approach to documenting collected data.

We expect the compilation of articles would enhance understanding of research trends and therapeutic applications making it a foundational resource for future studies. Additionally, by highlighting the most frequently studied species, such as *Nigella sativa* L. (Ranunculaceae)*, Rosmarinus officinalis* L. (Lamiaceae), *Salvia fruticosa* Mill. (Lamiaceae)*, Teucrium polium* L. (Lamiaceae), and *Thymus vulgaris* L. (Lamiaceae), we provide insights into which plants species have been prioritized for research. Furthermore, by categorizing dominant therapeutic uses, including cancer, bacterial infections, inflammation, diabetes, and fungal infections, we offer a roadmap for prioritizing future research collaborations across the region. The low number of ethnobotanical field studies (14%) we found compared to laboratory-based research (86%) underscores the need for more extensive documentation of traditional healing practices before they are lost. This finding should encourage future collaborations between researchers and local communities, ensuring that traditional knowledge is preserved and scientifically validated. Moreover, by identifying medicinal plants at risk due to overharvesting, such as *T. polium* L. (Lamiaceae) and *Origanum syriacum* L. (Lamiaceae), our findings play a supporting role for advocating for conservation policies and sustainable cultivation programs to protect biodiversity and maintain the availability of these valuable resources. On a policy level, we provide actionable recommendations for integrating traditional medicinal plants into modern healthcare systems. This includes developing standardized guidelines for safe medicinal plant use, implementing pharmacovigilance programs to monitor herb-drug interactions, and promoting community-based conservation programs to ensure sustainability. These efforts are essential for ensuring that medicinal plant research translates into practical healthcare applications while safeguarding regional biodiversity. Furthermore, our research is pioneering in its use of AI (GPT-4 Turbo) for large-scale text extraction in ethnopharmacology. By demonstrating the potential of AI to accelerate literature reviews and trend analysis, we pave the way for more efficient and systematic data processing in future medicinal plant research.

## 2 Methodology

**FIGURE 1:**
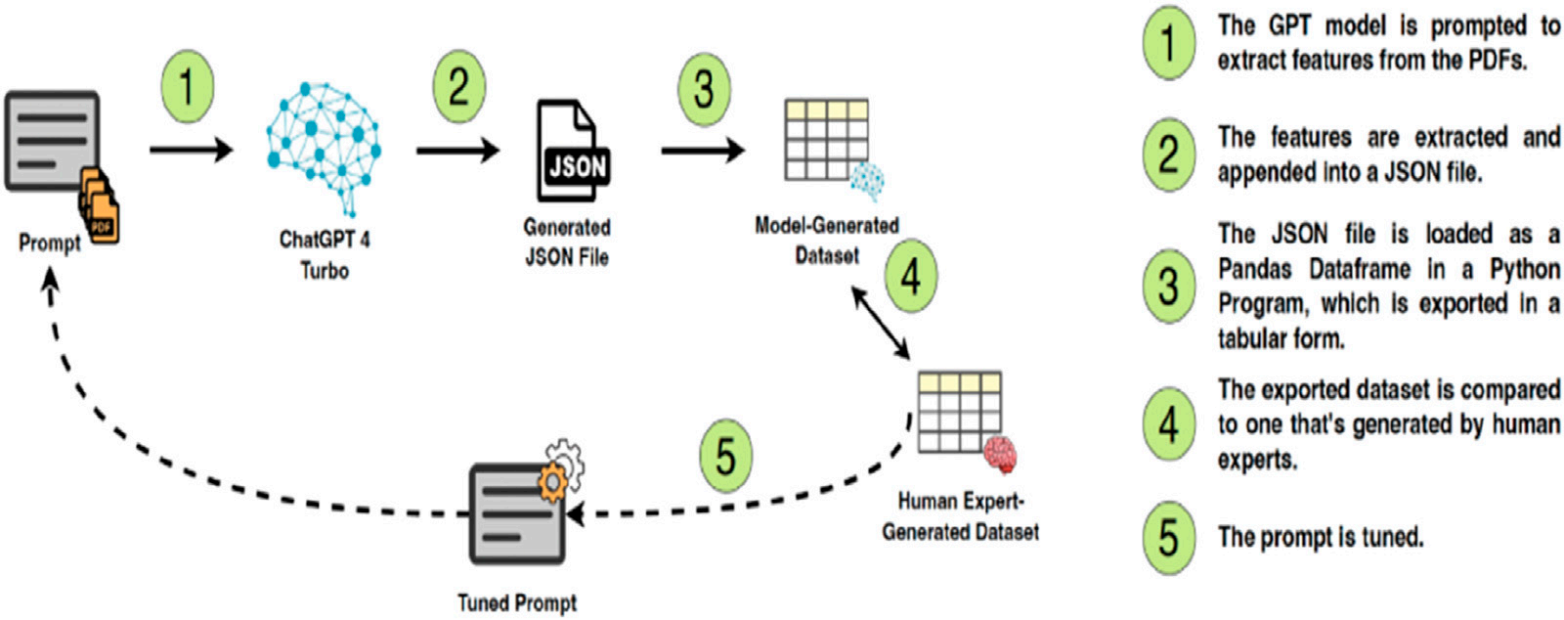
Extraction process roadmap.

The scoping review was designed and conducted following the Preferred Reporting Items for Systematic Review and Meta-Analysis (PRISMA) guidelines (Figure 1). A comprehensive literature search was initiated on June 14–16, 2023, across multiple electronic databases, including Arab World Research Source: Al Masdar^®^ (EBSCO), CABI Digital Library^®^, Iraqi Academic Scientific Journals^®^, MEDLINE^®^ (Ovid), Scopus^®^, and Web of Science Core Collection^®^ and Google Scholar^®^. To capture new publications, email alerts were set up in databases supporting this feature, covering updates until June 2024. The search strategy incorporated keywords related to medicinal plants and their associated concepts (e.g., herbal medicine, traditional medicine, phytotherapy), as well as geographic terms relevant to the Fertile Crescent (Iraq, Jordan, Lebanon, Palestine, and Syria). Full search strategies for each database are detailed in Supplementary File 1. Eligible sources comprised peer-reviewed journal articles, conference papers, and dissertations on medicinal plants used in the Fertile Crescent. Only publications in English or Arabic were considered, while studies lacking scientific plant names or containing incomplete data were excluded. No restrictions were applied to publication year, and all relevant documents available up to June 2024 were included. A total of 10,067 records were retrieved across all sources and were imported into reference management software (EndNote) for deduplication and screening. After removing duplicates, 6,038 unique records remained. An additional 104 records were identified through e-mail alerts, bringing the final dataset to 6,142 records for screening. Two investigators (Al-Sammarraie RN and Naalbandian S) independently reviewed titles and abstracts for relevance, followed by a full-text assessment of potentially eligible studies based on predefined criteria. Any discrepancies were resolved through discussion or consultation with a third reviewer (Talhouk SN). The two-stage screening process identified a total of 1,990 publications deemed relevant for data extraction. Quality assessment was performed based on relevance to the research scope, study rigor, and completeness of reported findings. Scientific names were standardized using the Medicinal Plant Names Services (MPNS) and Plants of the World Online (POWO) to ensure taxonomic accuracy.

To process a large volume of research efficiently, data extraction was performed using Generative Pretrained Transformer 4 (GPT-4) Turbo, a large language model (LLM) developed by OpenAI. The model was not fine-tuned on a custom dataset but was instead prompted iteratively using a structured approach to ensure optimal performance (Huang et al., 2024). LLMs leverage attention, deep learning architectures, and massive datasets that are in conversational AI applications and can generate human-like natural language text based on prompts. It is one of the largest and most powerful language models available, with 175 billion parameters (Jupudi, 2018; Sharma, 2024). In this study, OpenAI’s ChatGPT 4 Turbo is employed to extract key features from the comprehensive dataset of research publications using identifying key features, including scientific names of plants, reported therapeutic applications, type of research (laboratory-based *versus* field-based), and any pharmacological properties mentioned.

After applying the inclusion/exclusion criteria, the dataset comprised 1,990 research papers, ranging in length from 1 to 705 pages, all written in English, with publication dates spanning from 1958 to 2024. The extracted features included the title of the paper, the country of origin of the plant, the type of research conducted, the name of the medicinal plant discussed, whether the plant is traditional or non-traditional, its status as native or indigenous, its toxicity, and whether the plant was used as a treatment for various conditions such as skin disorders, respiratory problems, liver function improvement, and snake bites. The roadmap of the PDF feature extraction process is visualized in Figure 1.

In the initial phase, features were extracted from 300 PDFs. Each PDF was processed individually using a unified prompt. The extracted features were then compared with those manually determined by human experts. This comparison enabled several iterations of prompt refinement and tuning to ensure the model’s outputs closely aligned with the human-generated features. The extracted features were then loaded into a Pandas DataFrame in Python program for data analysis. Python is a high-level, interpreted, multi-purpose programming language that can be used for many applications that include statistical computing with various packages and functions (Kiranbala Nongthombam, 2021). The program was designed to process the extracted information, generate interpretations and analyses aligned with the objectives of the scoping review, and create visual representations of the data. These analyses provided valuable insights into research trends and the characteristics of medicinal plants in the Fertile Crescent. The final results were reviewed by human experts before inclusion in the database.

### 2.1 Potential biases and limitations

The integration of AI into literature reviews offers notable advantages, such as increased efficiency in data processing and synthesis. However, it also introduces several challenges that researchers must navigate to maintain the integrity and quality of their work. A prominent concern is the phenomenon known as “hallucination,” where AI systems generate information that appears plausible but is incorrect or misleading (Mostafapour et al., 2024). This occurs because AI models, despite their advanced capabilities, lack genuine understanding and rely solely on patterns learned from data. Therefore, despite the advantages of integrating AI into literature review, human validation is still needed to correct minor errors in plant classification or therapeutic claims. Moreover, AI models are limited by their training data and may struggle with nuanced ethnobotanical terminology or context-specific meanings. In addition, LLMs struggle in handling non-English languages that is why studies in non-English languages were excluded potentially omitting relevant findings.

## 3 Results and discussion

**FIGURE 2:**
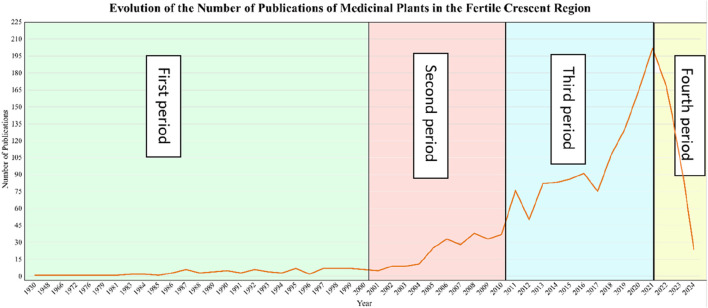
Temporal evolution of medical plants publications in the Fertile Crescent.

Figure 2 illustrates the temporal evolution of research publications on medicinal plants in the Fertile Crescent region from 1930 to 2024. We identified four distinct periods and explored the possible scientific, historical, and socio-political drivers behind the observed trends in research output.

From 1930 to 1999, medicinal plant research in the Fertile Crescent was low with fewer than 15 publications per year. This is probably because medicinal plant knowledge in the region was transmitted through oral traditions or written as Arabic compendiums rather than formal academic Eurocentric publications (Azaizeh et al., 2006). In addition, this period witnessed the end of European colonialism and was faced with economic instability, and political conflicts (Qassrawi, 2024). Additionally, during much of the 20th century, Western biomedicine dominated healthcare systems, undermining the value of traditional medicine and ethnobotanical research (World Health Organization, 2002).

A slight increase in publication numbers is observed after 2000, indicating a growing but still moderate interest in medicinal plant research. This shift aligns with the global rise of ethnopharmacology as an academic discipline and the World Health Organization’s renewed efforts to integrate traditional medicine into healthcare systems (World Health Organization, 2002). The establishment of research institutions focusing on medicinal plants, such as the Lebanese University’s Faculty of Pharmacy and Jordan University of Science and Technology’s medicinal plant research programs, may have played a role in increasing publications during this time. Technological advancements also contributed to this trend. The early 2000s saw increased accessibility to scientific journals through digital platforms, enabling greater knowledge exchange (Haleem et al., 2022). Additionally, increased interest in biodiversity conservation, such as the Global Strategy for Plant Conservation (GSPC) initiated in 2002, encouraged more research on medicinal flora (CBD, 2002). Despite this progress, the political instability in Iraq, Syria, and Palestine limited scientific advancements in the region. The Iraq War (2003) and ongoing conflicts in Palestine and Lebanon resulted in funding cuts and displacement of researchers, which may have slowed the growth of medicinal plant research.

Between 2010 and 2020, the number of publications surged, reflecting a strong global and regional shift toward natural product research and integrative medicine. Several factors contributed to this trend. These include scientific advancements in Medicinal Plant Research, the development of high-performance liquid chromatography (HPLC), mass spectrometry (MS), and DNA barcoding allowed for more precise identification of medicinal plant compounds, leading to an increase in phytochemical and pharmacological studies (Ganzera and Sturm, 2018). The rise of bioinformatics tools and network pharmacology further enabled researchers to explore plant-based drug discovery (Hopkins, 2008). Research funding for alternative medicine and biodiversity conservation increased significantly in the 2010s, as organizations such as the International Union for Conservation of Nature (IUCN) and United Nations Development Programme (UNDP) supported projects on medicinal plant sustainability in the Fertile Crescent (International Union for Conservation of Nature, 2017). The Convention on Biological Diversity (CBD) Nagoya Protocol (2010) also encouraged fair and equitable sharing of benefits from medicinal plant resources, further driving research efforts (Convention on Biological Diversity, 2010). A growing global interest in natural therapies, dietary supplements, and herbal medicine-based treatments led to increased market demand, prompting more scientific investigations (Ekor, 2014). This period saw an expansion of herbal medicine industries in Jordan, Lebanon, and Iraq, where pharmaceutical companies began incorporating plant-based compounds into commercial products (Azaizeh et al., 2006). The recognition of habitat loss and climate change impacts on medicinal plants became a pressing issue, resulting in more ecological studies focused on plant conservation in Lebanon’s Bekaa Valley, Jordan’s Dana Biosphere Reserve, and Iraq’s Mesopotamian wetlands (IUCN, 2018). These efforts translated into higher research output during this period.

A sharp spike in publications between 2020 and 2022 coincides with the COVID-19 pandemic, during which interest in plant-based antiviral compounds skyrocketed. Studies explored medicinal plants with antiviral, immune-boosting, and anti-inflammatory properties, leading to a surge in research output (Benarba and Pandiella, 2020). Countries in the Fertile Crescent, particularly Iraq, Jordan, and Lebanon, saw increased government and institutional funding for herbal medicine research aimed at identifying potential plant-derived treatments for COVID-19 (Ang et al., 2020). However, a steep decline after 2022 suggests a shift in research priorities post-pandemic. As COVID-19 research funding subsided, scientists may have redirected their focus toward other pharmaceutical and medical research areas. Another possible explanation for this decline is the saturation of herbal medicine publications during the pandemic, leading to fewer novel studies being conducted. The economic downturn in several Fertile Crescent countries post-pandemic may have also reduced funding for medicinal plant research, contributing to the sharp drop. The drop in 2023–2024 may also be due to incomplete data collection or publication delays, as the research only includes publications up until June 2024.

With respect to plants, the review recorded 1,141 medicinal plant species in the Fertile Crescent. This number is significant when compared with the number of medicinal plant species reported in China and India, the two leading countries in terms of traditional knowledge and research on medicinal plants. Both India and China have exceptionally rich medicinal plant diversity, with India having around 7,500 medicinal plant species and China having over 11,000 species (Huang et al., 2024; Singh et al., 2022; Xiong et al., 2020). In Table 1 we show how the Fertile Crescent compares favorably with these two countries showing the richness in diversity of medicinal plants in the region.

**TABLE 1:** The number of medicinal plant species in India, China, and the Fertile Crescent.

Countries	Number of medicinal plant species	Unit area in square kilometers	Number of medicinal plant species per unit area (square kilometer)
India	7,500	3,287,263	438
China	11,146	9,596,961	861
Fertile Crescent	1,141	1,021,452	895

Globally, it is estimated that there are between 72,000 and 77,000 medicinal plant species, which represent around 17%–18% of the world’s flora (Chekole et al., 2015; Hazarika et al., 2023; Tshabalala et al., 2022). China, Japan, India, and the USA are among the top countries contributing to research on traditional herbal medicine (Hazarika et al., 2023; Pironon et al., 2024).

Despite the large number of recorded medicinal plant species in the Fertile Crescent, the review sheds light on the fact that each country seems to focus on the most popular medicinal plant species locally. So, although the top research plants species are native to all five countries the priorities do not seem aligned between countries. Instead, researchers in Iraq focus on *Nigella sativa* L. (Ranunculaceae), *Rosmarinus officinalis* L. (Lamiaceae) is Jordan’s focus, *Teucrium polium* L. (Lamiaceae) in Palestine, *Peganum harmala* L. (Nitrariaceae) in Syria, and *Origanum syriacum* L. (Lamiaceae) in Lebanon (Table 2).

**TABLE 2:** Most investigated medicinal plant species in the Fertile Crescent and their reported traditional uses.

Medicinal plant species	Country	Reported uses
*Nigella sativa* L. (Ranunculaceae)	Iraq	Immune system effects Skin disorders Respiratory problems
*Rosmarinus officinalis* L. (Lamiaceae)	Jordan	Cardiovascular system effects Skin disorders Respiratory problems Liver function Kidney function Lipid panel
*Teucrium polium* L. (Lamiaceae)	Palestine	Urinary tract infections Gastrointestinal problems
*Peganum harmala* L. (Nitrariaceae)	Syria	Nervous system effects
*Origanum syriacum* L. (Lamiaceae)	Lebanon	Gastrointestinal problems

Further elaboration about published research on these species (Table 2) shows that *Nigella sativa* L. (Ranunculaceae) is investigated in Iraq for its effects on the immune system, skin disorders, and respiratory problems. *Nigella sativa* L. (Ranunculaceae) is well known to the Arab World and to Islam where it is described as a “*cure for every disease, except death*.” (Sahih al-Bukhari, Imam Muhammad al-Bukhari (870 AD). Numerous studies have demonstrated its ability to enhance the immune system by modulating cytokine production, increasing phagocytic activity, and stimulating the proliferation of immune cells (Abdullah et al., 2022; Al Turkmani et al., 2015). The second most reported uses are for skin disorders and respiratory problems, which can also be attributed to the plant’s anti-inflammatory, antioxidant, and anti-microbial activities that are beneficial for these conditions ((Alharchan and Ashor, 2010) Aqel, 1991; Osman and Methil Kannan, 2013). *Nigella sativa* L. (Ranunculaceae) contains active compounds including thymoquinone, thymohydroquinone, dithymoquinone, thymol, and carvacrol. These compounds exhibit immunomodulatory, anti-inflammatory, and anti-microbial properties, which could explain the reported research on *N. sativa* L. (Ranunculaceae) in Iraq (Niu et al., 2021; Wei et al., 2022).

In Jordan, *Rosmarinus officinalis* L. (Lamiaceae) has been traditionally used to improve blood circulation and heart health (Khatib et al., 1998), and research has demonstrated that it possesses cardioprotective properties due to its ability to modulate lipid profiles, reduce blood pressure, and improve endothelial function. Other frequently reported uses of *R. officinalis* L. (Lamiaceae) are for skin disorders, respiratory problems, liver function, kidney function, and lipid panel, which could be explained by the plant’s rich pharmacological activities, including anti-inflammatory, antioxidant, hepatoprotective, and hypolipidemic effects (Abu-Al-Basal, 2010; Umran et al., 2013). Carnosic acid, carnosol, rosmarinic acid, ursolic acid, and caffeic acid are active compounds found in *R. officinalis* L. (Lamiaceae) (Borrás-Linares et al., 2014; Vieira et al., 2022). These compounds possess antioxidant, anti-inflammatory, and cardioprotective properties, which align with the reported uses of *R. officinalis* L. (Lamiaceae) for reported research in Jordan (Sharma et al., 2020; Vieira et al., 2022).

*Teucrium polium* L. (Lamiaceae) in Palestine is mainly reported for its urinary tract effects. This plant has been traditionally used in the Middle East to treat various urinary tract disorders, including urinary tract infections, kidney stones, and bladder problems (Al-Bahtiti, 2012). Another reported use is for gastrointestinal problems, which is also a common traditional application of this plant due to its anti-inflammatory, antispasmodic, and gastroprotective properties (Al-Bahtiti, 2012). *Teucrium polium* L. (Lamiaceae) contains flavonoids, terpenoids, and phenolic compounds. These compounds have anti-inflammatory, antioxidant, and antimicrobial properties, which could contribute to the reported research on *T. polium* L. (Lamiaceae) in Palestine (Alali and Khazem, 2020; Jaradat et al., 2017; Suleiman et al., 1988).

In Syria, the focus is on *Peganum harmala* L. (Nitrariaceae) a plant species that has been used in traditional medicine to treat a variety of neurological and psychiatric conditions, such as anxiety, depression, and epilepsy, due to its ability to modulate neurotransmitter systems and exert neuroprotective effects (Asgarpanah and Ramezanloo, 2012; Shatarat et al., 2020). Harmine, harmaline, harmalol, harmol, and peganine are active compounds found in *P. harmala* L. (Nitrariaceae). These compounds have been associated with neuroprotective, anti-depressant, and anxiolytic effects, which might explain the reported research on *P. harmala* L. (Nitrariaceae) in Syria (Doskaliyev et al., 2021b; Wang et al., 2022).

Lastly, *Origanum syriacum* L. (Lamiaceae) in Lebanon is investigated for its traditional use in the Fertile Crescent to treat various gastrointestinal disorders, such as indigestion, diarrhea, and stomach ulcers, which may be because of its anti-microbial, anti-inflammatory, and antioxidant properties (Al Hafi et al., 2016; AlKahlout et al., 2022; Daouk et al., 1995; Mesmar, Abdallah, Hamade, et al., 2022). *Origanum syriacum* L. (Lamiaceae) contain carvacrol, thymol, rosmarinic acid, and quercetin. These compounds possess anti-microbial, anti-inflammatory, and antioxidant properties, which could underline the reported research on *O. syriacum* L. (Lamiaceae) in Lebanon (El-Moneim et al., 2014; Mesmar, Abdallah, Badran, et al., 2022).

**FIGURE 3:**
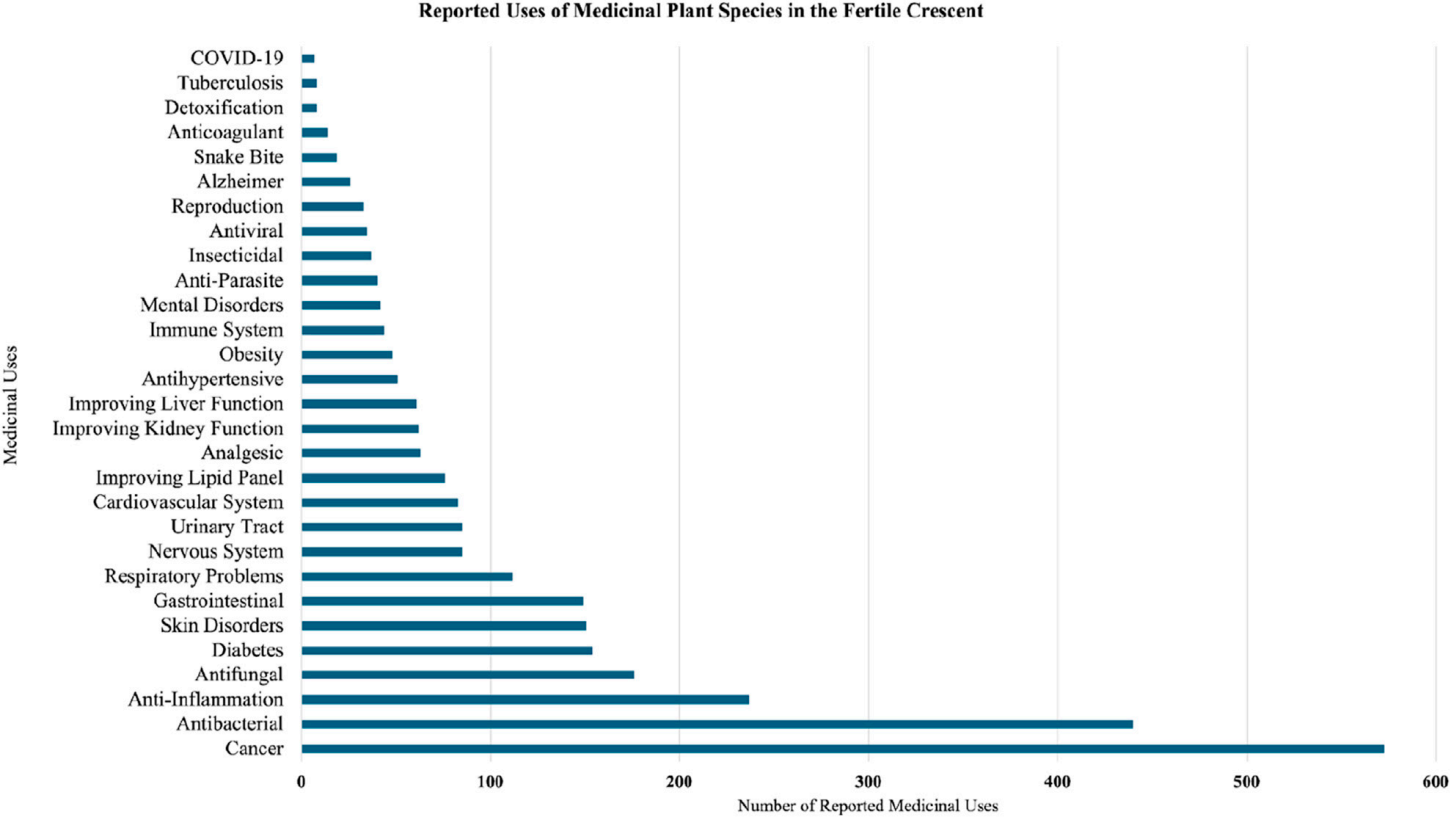
Potential pharmacological uses of medicinal plants in the Fertile Crescent.

The review revealed 29 medical conditions under investigation in the Fertile Crescent with the two most investigated are cancer (573 mentions) and bacterial infections (440 mentions). These were followed by 27 health conditions including inflammation (237), fungal infections (176), diabetes (154), skin disorders (151), gastrointestinal (149), respiratory (112), urinary tract (85), nervous system (85), cardiovascular (83), lipid panels (76), analgesics (63), kidney function (62), liver function (61), hypertension (51), obesity (48), immune system (44), mental health (42), parasites (40), insecticidal (37), viral (35), reproductive health (33), Alzheimer’s (26), snake bite (19), anticoagulant (14), detoxification (8), tuberculosis (7), and COVID-19 (8) (Figure 3).

### 3.1 Anti-cancer properties

The exploration of plants species with anti-cancer properties in all biomes and ecosystems, including the Fertile Crescent, is still needed as it offers potential alternatives to conventional cancer treatments, which can have significant side effects and may not be effective for all types of cancer (Newman and Cragg, 2020; Yan et al., 2023). The anti-cancer properties of medicinal plants under investigation in the Fertile Crescent countries are thought to be due to their ability to modulate multiple cellular pathways involved in cancer development and progression. Some plant species were reported to help prevent cancer initiation and progression through their anti-inflammatory and antioxidant effects, while others report anti-proliferative and pro-apoptotic effects, which can help inhibit cancer cell growth and induce cell death (Samaha et al., 2019).

Nine plant species have been investigated *in vitro* and *in vivo* for their anticancer properties in the Fertile Crescent; these include *R. officinalis* L. (Lamiaceae), *N. sativa* L. (Ranunculaceae), *Salvia fruticosa* Mill. (Lamiaceae), *T. polium* L. (Lamiaceae), *Thymus vulgaris* L. (Lamiaceae), *Crocus sativus* L. (Iridaceae), *O. syriacum* L. (Lamiaceae), *Lavandula stoechas* L. (Lamiaceae), and *Satureja thymbra* L. (Lamiaceae). The most investigated species is *N. sativa* L. (Ranunculaceae), which contains in its seeds thymoquinone, a metabolite that has been shown to inhibit the growth of cancer cells and induce apoptosis in various cancers, including breast, lung, and colon cancer (Alabdallat and Alanazi, 2023; Dabrowski et al., 2024; Yaghi et al., 2023). Thymoquinone has also been shown to inhibit the expression of genes involved in cancer cell proliferation and survival, including cyclin D1 and Bcl-2. Similarly, *Salvia viscosa* Jacq. (Lamiaceae), which contains in its essential oils a metabolite called caryophyllene oxide, has been shown to inhibit cancer cell growth and induce apoptosis in various types of cancer, including colon and prostate cancer by inhibiting gene expression linked to cancer cell proliferation and survival, including cyclin D1 and Bcl-2 (Russo et al., 2018). Plant extracts with anti-proliferative and pro-apoptotic effects were also reported for *T. polium* L. (Lamiaceae) on human prostate cancer cells, *O. syriacum* L. (Lamiaceae) on human ovarian cancer cells, and *S. thymbra* L. (Lamiaceae) on human stomach cancer cells (Atoum et al., 2023; Tabaza and Aburjai, 2024).

Essential oils from various species were also found to inhibit cancer cell growth and induce apoptosis including *R. officinalis* L. (Lamiaceae) on breast cancer cells, *S. fruticosa* Mill. (Lamiaceae) on colon cancer cells, *L. stoechas* L. (Lamiaceae) on liver cancer cells, and *T. vulgaris* L. (Lamiaceae) on skin cancer cells.

### 3.2 Anti-bacterial effects

The Arab region is facing a significant rise in anti-bacterial resistance (Alkheraije, 2024; Ballouz et al., 2021) due to the availability of antimicrobials over the counter, inadequate infection prevention and control programs, and the presence of poor-quality antibiotics in the market, especially in conflict zones (Abdallah et al., 2023; Ballouz et al., 2021; Hamarsheh et al., 2021; Rizk et al., 2021). More specifically, studies conducted in countries of the Fertile Crescent have noted an alarming increase in multidrug-resistant bacteria, including strains resistant to last-line antibiotics (Abdel-Massih and El Beyrouthy, 2022) like ESKAPE organisms (*Acinetobacter* baumannii, *Pseudomonas aeruginosa*, and *Klebsiella* pneumonia) (Morel et al., 2021; Selvarajan et al., 2022; Wang et al., 2022). On the other hand, medicinal plants in the Arab world, including the Fertile Crescent region, offer a vast array of bioactive metabolites that may combat bacterial infections effectively (Darwich et al., 2022; Roula and El Beyrouthy, 2022). Our review revealed that the anti-bacterial properties of plant species investigated in the Fertile Crescent are attributed to their ability to inhibit bacterial growth, prevent biofilm formation, and induce bacterial cell death. One of the most studied species for its anti-bacterial properties is *T. vulgaris* L. (Lamiaceae) (Hudaib and Aburjai, 2007; Shalaal et al., 2019). This plant’s essential oil contains high levels of a metabolite called thymol, which has been shown to inhibit the growth of various types of bacteria, including *Staphylococcus aureus* and *Escherichia coli*. Thymol has also been shown to prevent biofilm formation and induce bacterial cell death. The essential oil of *O. syriacum* L. (Lamiaceae) contains a metabolite called carvacrol which has been shown to inhibit the growth of various types of bacteria, including *P. aeruginosa* and *Klebsiella pneumonia* (Mesmar et al., 2022; Mohamad et al., 2021; Shehadeh et al., 2019). Carvacrol has also been observed to prevent biofilm formation and induce bacterial cell death.

The over-representation of cancer and bacterial infection studies (51% combined) may reflect global funding priorities as well as the pressing healthcare needs of the region. While antimicrobial resistance (AMR) is a well-documented global crisis (Ballouz et al., 2021), conditions such as cardiovascular diseases and respiratory disorders, which are prevalent in the Middle East, remain under-researched in ethnopharmacology. This suggests an opportunity for future research to realign priorities with regional health burdens.

### 3.3 Anti-inflammatory properties

The anti-inflammatory properties of medicinal plants investigated in countries of the Fertile Crescent region are thought to be due to their ability to modulate multiple cellular pathways involved in inflammation. For example, some plant species have been found to have antioxidant effects, which can help reduce oxidative stress and inflammation. Others have been revealed to have immunomodulatory effects, which can help regulate the immune response and prevent excessive inflammation. Plant species found to possess anti-inflammatory properties, include *N. sativa* L. (Ranunculaceae)*, R. officinalis* L. (Lamiaceae)*, S. fruticosa* Mill. (Lamiaceae)*, T. polium* L. (Lamiaceae)*, T. vulgaris* L. (Lamiaceae)*, O. syriacum* L. (Lamiaceae)*, Lavandula coronopifolia, S. thymbra* L. (Lamiaceae), and *Achillea Fragrantissima* (Forssk.) Sch. Bip. (Asteraceae) (Abdel-Massih and Abraham, 2014; Aburjai et al., 2005; Al-Tawarah, 2022; Kharma and Hassawi, 2006; Marrelli et al., 2018; Merajuddin et al., 2019; Naseef et al., 2022; Sadeq et al., 2021). These plant species contain various bioactive metabolites, including flavonoids, phenolic acids, and terpenes, which have been demonstrated to have anti-inflammatory activities *in vitro* and *in vivo* (Bahramikia and Yazdanparast, 2012; Rafieian-Kopaei et al., 2014). Evidence of anti-inflammatory effects using animal models of inflammation was attributed to the inhibition of inflammatory cytokines. This was reported for plant extracts of *N. sativa* L. (Ranunculaceae), *S. fruticosa* Mill. (Lamiaceae), *O. syriacum* L. (Lamiaceae), *Achillea millefolium* L. (Asteraceae), *Melissa officinalis* L. (Lamiaceae), and *T. polium* L. (Lamiaceae) (Aldal’in, 2018; Salamon et al., 2019). Essential oils of *R. officinalis* L. (Lamiaceae), *T. vulgaris* L. (Lamiaceae), *Cinnamomum verum* J. Presl (Lauraceae), *Punica granatum* L. (Lythraceae), and *L. coronopifolia* poir. (Lamiaceae) were found to inhibit the production of inflammatory mediators (Bakkour et al., 2011; Naseef et al., 2022; Hekmat and Al-Obeidi, 2019). In addition, plant species with anti-inflammatory properties in their underground organs (roots and rhizomes) include *Glycyrrhiza glabra* L. (Fabaceae) roots, which contain glycyrrhizin, and *Zingiber officinale* Roscoe (Zingiberaceae) rhizomes, which contain gingerol. Both metabolites have been reported to inhibit the production of pro-inflammatory cytokines and enzymes, stimulate the production of anti-inflammatory cytokines, and suppress the activation of inflammatory cells, such as macrophages and T cells (Abu-Al-Basal, 2010; Umran et al., 2013).

### 3.4 Anti-fungal characteristics

One of the most studied medicinal plant species in the Fertile Crescent for anti-fungal properties is *Allium sativum* L. (Amaryllidaceae) whose bulbs contain allicin, a metabolite shown to prevent biofilm formation, induce fungal cell death, and inhibit the growth of various types of fungi, including *Candida albicans* and *Aspergillus fumigatus* (Abdulkhaleq et al., 2022; Abdulla and Ismael, 2023; Fahed et al., 2021). Studies have also investigated the bark of *C. verum* J. Presl (Lauraceae) which contains cinnamaldehyde, a metabolite that has also been observed to prevent biofilm formation and induce fungal cell death (Ubaid et al., 2022) and to inhibit the growth of various types of fungi, including *C. albicans* and *A. fumigatus* (Al-Zereini et al., 2022). Other plant species extracts reported to possess anti-fungal properties include extracts of *T. vulgaris* L. (Lamiaceae) which was tested against *C. albicans* and *Aspergillus flavus* (Al-Saidy et al., 2012; Azeez, 2020), *Teucrium Polium* L. which was tested against *C. albicans* and *Candida tropicalis* (Abdullah et al., 2022; Al-Bahtiti, 2012; Darwish and Aburjai, 2011; Rahmouni et al., 2021), *Origanum* syriacum L. (Lamiaceae) which was tested against *C. albicans* and *Candida krusei* (Al Hafi et al., 2016; Daouk et al., 1995; Kassaify et al., 2008; Mesmar et al., 2022), *S. thymbra* L. (Lamiaceae) which was tested against *C. albicans* and *Candida glabrata* (Al Hafi et al., 2017; Beyrouthy et al., 2013), and *M. officinalis* L. (Lamiaceae) which was tested against *C. albicans* and *C. tropicalis* (Aldal’in, 2018).

### 3.5 Anti-diabetic properties

Many plant species in the Fertile Crescent were shown to possess anti-diabetic properties because of their ability to increase insulin secretion, improve insulin sensitivity, and reduce glucose absorption in the gut. For example, extracts of *N. sativa* L. (Ranunculaceae), *T. polium* L. (Lamiaceae), and *A. millefolium* L. (Asteraceae) reduced blood glucose levels and improved insulin sensitivity in streptozotocin-induced diabetic rats (Fadheel, 2019; Mosleh et al., 2022; Raziani et al., 2022). Extracts of *C. verum* J. Presl (Lauraceae) reduced blood glucose levels and improved insulin sensitivity in alloxan-induced diabetic rats (Alkubaisy et al., 2019). The metabolite 4-hydroxyisoleucine found in *Trigonella foenum-graecum* L. (Fabaceae) seeds was shown to inhibit glucose absorption, stimulate insulin secretion, improve insulin sensitivity, and reduce blood glucose levels in diabetic patients (Abdel-Barry et al., 1997; Abdel-Barry et al., 2000).

### 3.6 Other medicinal plant properties

This section tackles the smaller number of studies conducted to address various ailments and disorders. Research on the beneficial effects of plant species extracts on ***skin health*** is reportedly related to the plants’ abilities to inhibit inflammation, prevent bacterial growth, and improve skin hydration. Aloin found in the gel of *Aloe vera* (L.) Burm. f. (Asphodelaceae), and calendulin which is present in the flowers of *Calendula officinalis* L. (Asteraceae) have both been shown to inhibit inflammation, prevent bacterial growth, improve skin hydration, and reduce skin disorders such as acne and eczema (Hasan and Abdullah, 2022). Research has also confirmed traditional uses of plant species for skin health, these include extracts of *Myrtus communis* L. (Myrtaceae), *P. granatum* L. (Lythraceae), and *S. fruticosa* Mill. (Lamiaceae) (Abu-Darwish et al., 2013). Studies reported that these plants possess anti-inflammatory, anti-bacterial, and antioxidant properties, attributing their effect on the skin by reducing inflammation, preventing infection in skin wounds, and promoting wound healing (Al-Mariri et al., 2016; Barzani et al., 2014; Hamidi et al., 2023).

Many plant species have been traditionally used by peoples of the Fertile Crescent for centuries to treat various types of ***gastrointestinal disorders*** including irritable bowel syndrome, inflammatory bowel disease, and gastroesophageal reflux disease. More specifically, glycyrrhizin found in the roots of *G. glabra* L. (Fabaceae) and gingerol found in the rhizomes of *Z. officinale* Roscoe (Zingiberaceae) have both been shown to inhibit inflammation, prevent bacterial growth, improve gut motility, and reduce the severity of gastrointestinal disorders such as irritable bowel syndrome (Abbas, 2020; Al-Mousawi et al., 2022; Shawarb et al., 2021). Cuminum *cyminum* L. (Apiaceae) and *Foeniculum vulgare* Mill. (Apiaceae), were reported to alleviate symptoms associated with irritable bowel syndrome by reducing inflammation (Bouhenni et al., 2021; Karik et al., 2021; Kashamar et al., 2018). Other medicinal plants in the Fertile Crescent, such as *Trachyspermum ammi* (L.) Sprague (Apiaceae) and *Carum carvi* L. (Apiaceae), were reported to have carminative and anti-spasmodic properties, making them effective in the treatment of digestive disorders such as bloating, cramps, and diarrhea (Abd Al-Behadili et al., 2019; Anwar et al., 2016; Naquvi et al., 2022). Furthermore, the following species, *Pimpinella anisum* L. (Apiaceae), *Coriandrum sativum* L. (Apiaceae), and *Achillea* L. (Asteraceae) (Kharma and Hassawi, 2006), traditionally used for gastrointestinal disorders were observed to have anti-inflammatory and antioxidant properties, reducing inflammation and promoting gut health, and modulate the gut microbiota (Al-Bayati, 2008; Al-Daody and Al-Ta’ee, 2018; Al-wendaw et al., 2021; Sihoglu Tepe and Tepe, 2015; Sulaiman and Ahmed, 2018).

Many plant species in the Fertile Crescent are aromatic, a trait that is essential for their survival and defense during hot dry summers. These same species have also been traditionally used by people in the region to treat ***respiratory illnesses***. For example, studies indicated that the essential oil of *T. vulgaris* L. (Lamiaceae) which contains thymol, inhibits inflammation and prevents bacterial growth (Al-Assaf et al., 2023; Al-Saidy et al., 2012; Aziz et al., 2022; Shalaal et al., 2019). Thymol has also been demonstrated to improve lung function, have expectorant properties, help relieve coughs and congestion, and reduce the severity of respiratory disorders such as asthma (Al Hafi et al., 2017; Alimari et al., 2023). Similarly, *Eucalyptus globulus* Labill. (Myrtaceae) and *R. officinalis* L. (Lamiaceae), have decongestant and anti-inflammatory properties, making them effective in the treatment of respiratory disorders such as colds, flu, and sinusitis (Abdel-Massih and Abraham, 2014; Al-Taai et al., 2022; Aqel, 1991; Dheyab et al., 2022; Qabaha et al., 2016). *Mentha × piperita* L. (Lamiaceae), was reported to reduce inflammation and alleviate symptoms associated with bronchitis and asthma (Al-Saidy et al., 2012; Shawarb et al., 2021). *Glycyrrhiza glabra* L. (Fabaceae), and *P. granatum* L. (Lythraceae) were reported to have anti-inflammatory and antioxidant properties, reducing inflammation and promoting lung health (Al-Laham and Al-Fadel, 2013; Al-Mousawi et al., 2022; Husin et al., 2015; Upadhyay et al., 2020; Hekmat and Al-Obeidi, 2019). *Cinnamomum verum* J. Presl (Lauraceae) was reported to have bronchodilatory properties, relieving bronchospasms and improving lung function (Al-Zereini et al., 2022), while *Origanum majorana* L. (Lamiaceae), *and Lavandula angustifolia* Mill. (Lamiaceae), were reported as having expectorant and anti-inflammatory properties, relieving coughs and congestion, and reducing inflammation in the lungs (Abdel-Massih and Abraham, 2014; Ahmed et al., 2022; Koleilat et al., 2017; Marrelli et al., 2016; Raafat et al., 2013).

Most plant species traditionally used in the Fertile Crescent to treat ***urinary tract disorders***, have been found to have diuretic, anti-inflammatory, and anti-microbial properties, and were reported to inhibit bacterial growth, prevent stone formation, and improve urinary tract function (Ahmed et al., 2021; Akour et al., 2021; Allami et al., 2020). For example, Urticin found in *Urtica dioica* L. (Urticaceae) leaves inhibits bacterial growth, prevents urinary tract infections, prevents stone formation, and improves urinary tract function (Ahmed et al., 2021). Several other species investigated include *U. dioica* L. (Urticaceae), *Petroselinum sativum* Hoffm. (Apiaceae) (Kashamar et al., 2018), *Arctium lappa* L. (Asteraceae) (Al-Shammaa et al., 2013), *Taraxacum officinale* F.H.Wigg. (Asteraceae) (Ali et al., 2021), *Cichorium intybus* L. (Asteraceae) (Abdullah et al., 2019), *Juniperus phoenicea* L. (Cupressaceae) (Abu-Darwish et al., 2013), *Cynara scolymus* L. (Asteraceae) (Nasser, 2012), *Achillea fragrantissima* (Forssk.) Sch. Bip (Alsohaili, 2018), *F. vulgare* Mill. (Apiaceae) (Kashamar et al., 2018), and Rhus *coriaria* L. (Anacardiaceae) (Marouf et al., 2022).

People in the Fertile Crescent traditionally used an array of medicinal plant species with beneficial effects on ***nervous system health***. Research has shown that these plant species have anxiolytic, sedative, and neuroprotective properties thought to be due to their ability to modulate the activity of neurotransmitters, reduce oxidative stress and inflammation, and improve cognitive function (Abbas, 2020; Alsmadi et al., 2018). For example, *M. officinalis* L. (Lamiaceae) was reported to help reduce anxiety and promote relaxation through its anxiolytic and sedative properties (Aldal’in, 2018; Rasool and Muhammad, 2013). *Lavandula angustifolia* Mill. (Lamiaceae) has neuroprotective activity as it reduces oxidative stress and inflammation in the nervous system through aromatherapy (Koleilat et al., 2017). Bacoside, a metabolite found in the leaves of *Bacopa monnieri* (L.) Wettst. (Plantaginaceae) was shown to inhibit inflammation and prevent oxidative stress (Alwash, 2018). Bacoside has also been shown to improve neurotransmitter function and reduce the severity of nervous system disorders such as anxiety and depression (Al-Snafi, 2015). *Crocus sativus* L. (Iridaceae) was reported to have neuroprotective and anti-inflammatory properties, making it effective in the treatment of neurodegenerative diseases such as Alzheimer’s and Parkinson’s (Makhlouf et al., 2011; Samaha et al., 2021). *Ferulago angulata* (Schltdl.) Boiss. (Apiaceae) (Bagci et al., 2016), *Salvia fructicosa* Mill. (Lamiaceae) (Abu-Darwish et al., 2013), and *T. vulgaris* L. (Lamiaceae) (Al-Assaf et al., 2023) were reported to have antioxidant, anti-inflammatory, anxiolytic, and sedative properties, which helped improve cognitive function and memory, reduce anxiety and promote relaxation. Other medicinal plant species, namely, *P. harmala* L. (Nitrariaceae), *O. syriacum* L. (Lamiaceae), were reported to have antidepressant and anxiolytic properties, making them effective in the treatment of depression and anxiety (Jasim, 2019; Kanaan et al., 2014).

Our review shows that the number of publications resulting from laboratory research dominates investigations (86%) while only a smaller proportion (approximately 14%) addresses field research. This noted lack of attention to traditional knowledge holders by researchers in the Fertile Crescent has been also observed in global ethnopharmacological research, where experimental studies often dominate over traditional knowledge documentation (Pirintsos et al., 2022). This lack of alignment between traditional and modern research may hinder the translation of findings into practical applications for local communities. Our findings are confirmed by a recent scoping review by Alzweiri et al. (2011); Abu-Odeh et al. (2023). The authors who focused on Jordanian medicinal plants and analyzed 124 articles published between 2000 and 2022 noted a predominance of laboratory-based studies over field research. In contrast, Astutik et al. (2019) reported a higher prevalence of field-based studies in South Asian countries, indicating stronger preservation of traditional knowledge transmission. Similarly, da Silva et al. (2018) found that greater collaboration exists between ethnobotanists and medical researchers in Latin America, leading to a higher percentage of clinical studies. This finding underscores the need for a more integrated research approach in the Fertile Crescent, bridging laboratory work with field-based ethnobotanical surveys.

### 3.7 Implications for practice and policy

Based on our analysis, we propose three key policy recommendations that are likely to be most effective in addressing the challenges of medicinal plant research and conservation in the Fertile Crescent region: First, for TAIM to become part of integrative medicine in the region, national regulatory bodies should establish guidelines for the safe use of traditional remedies, similar to efforts in China’s Traditional Chinese Medicine modernization initiative (Zhang et al., 2022; Wang et al., 2021). Pharmacovigilance programs should be developed to monitor potential herb-drug interactions. Second, ethnopharmacologists should engage with traditional healers in Fertile Crescent countries to document oral knowledge before it disappears by collectively creating a database of regional medicinal plants. Third, overharvesting of medicinal plants in the Fertile Crescent threatens biodiversity. Our review identified several species (e.g., *T. polium* L. (Lamiaceae), *O. syriacum* L. (Lamiaceae)) that are at risk due to unsustainable harvesting. Policymakers should implement community-based conservation programs to promote sustainable cultivation practices and share with communities the commercial benefits when they arise.

### 3.8 Limitations and future research directions

Despite its comprehensive scope, this study has several limitations. Only English studies were included, potentially omitting valuable research published in Arabic, Kurdish, Persian, or French. While GPT-4 Turbo improved efficiency, AI models may misinterpret context or fail to differentiate between ambiguous plant names. Manual validation reduces errors, but some misclassifications may persist. Additionally, a significant proportion of studies lacked phytochemical standardization, and only 20% included clinical or *in vivo* trials, affecting the generalizability of findings. Future research should aim to expand language coverage, implement human-AI hybrid screening processes, and prioritize clinical validation of promising plant extracts.

## 4 Conclusion

This review sheds light on the state of TAIM with a focus on the Fertile Crescent region. The findings revealed that the region harbors a rich diversity of medicinal plants and a clear inclination towards scientific validation of the medicinal properties of plants. This trend underscores the region’s commitment to evidence-based medicine and the rigorous examination of plant-derived metabolites for their potential therapeutic benefits (Dafni and Böck, 2019; Talib et al., 2020). The high percentage of laboratory research also highlights regional interest in phytochemical analysis, signaling a nuanced understanding of the bioactive metabolites present in medicinal plants and their mechanisms of action (Talib et al., 2020). On the other hand, research on traditional use of medicinal plants accounted for approximately 14% of the total research conducted on medicinal plants in the Fertile Crescent. This suggest that scientific research does not seem to be harnessing traditional knowledge and practices which remain integral to the region’s healthcare landscape, there is a notable shift towards incorporating scientific methodologies and experimental approaches in understanding and harnessing the medicinal properties of indigenous flora (Dar-Odeh and Abu-Hammad, 2020; Dehyab et al., 2020). Although the predominance of experimental research in medicinal plant studies aligns with global trends in evidence-based healthcare practices and fostering innovation in natural product research (Baydoun et al., 2015; Saad et al., 2005), the lack of field research may lead to loss of traditional plant knowledge that would go unrecorded. The documentation of the traditional distribution and uses of medicinal plants is crucial, given the reliance on complementary and alternative medicine in developing countries and the increasing threats to the natural habitats and conservation status of these valuable species. The establishment of a systematic database to record the medicinal plants of the Fertile Crescent is a step towards preserving this important aspect of the region’s collective cultural and ecological heritage. Given these insights, the review points to the need for supporting collaborations between traditional healers, ethnobotanists, pharmacologists, and healthcare professionals to open an information exchange corridor between all stakeholders for the purpose of creating standardized methodologies and regulatory frameworks that facilitate the integration of traditional medicine into healthcare systems and support the documentation and preservation of traditional knowledge before it disappears. Future research focus should also support the development of the sector by expanding clinical trials to validate the efficacy and safety of commonly studied medicinal plants and developing pharmacovigilance programs to monitor herb-drug interactions and ensure consumer safety.
