# Supplementary material for: An artificial intelligence-aided scoping review of medicinal plant research in the Fertile Crescent
Source: Front Pharmacol. 2025 Jun 3;16:1542709. doi: 10.3389/fphar.2025.1542709 (PMC12171211; doi:10.3389/fphar.2025.1542709)
Supplement: Supplementary file 1 [file Table1.docx]

Medicinal Plants in the Fertile Crescent

Search Strategies

Summary by source:

| **Source** | **Date Searched** | **Number of Results (including duplicates)** | **Number of Results**  **(after removal of duplicates)** |
| --- | --- | --- | --- |
| Arab World Research Source: Al Masdar | June 14, 2023 | 216 | 142 |
| CABI Digital Library | June 14, 2023 | 3,456 | 2,206 |
| Iraqi Academic Scientific Journals | June 14-15, 2023 | 110 | 92 |
| MEDLINE | June 14, 2023 | 1,178 | 103 |
| Scopus | June 14, 2023 | 2,972 | 2,938 |
| Web of Science Core Collection | June 14, 2023 | 1,455 | 271 |
| Google Scholar | June 14-16, 2023 | 680 | 286 |
|  | **Total** | 10,067 | 6,038 |
| E-mail alerts* |  |  | 104 |
| **Total** | |  | **6,142** |

*104 articles were identified through e-mail alerts created while database searching.

1. Database searches:

| Database name: *Arab World Research Source: Al Masdar*  Database platform: *EBSCO*  Date searched: *June 14, 2023*  Limits applied: *Languages (English, Arabic)*  Alert set up: *Yes* | | |
| --- | --- | --- |
| **Search** | **Query** | **Number of Results** |
| S1 | TI ( ((plant OR eucalyptus OR rapeseed OR castor OR clove OR corn OR cottonseed OR croton OR iodized OR ethiodized OR linseed OR palm OR "rice bran" OR safflower OR sesame OR soybean OR sunflower OR "tea tree") N3 oil*) ) OR AB ( ((plant OR eucalyptus OR rapeseed OR castor OR clove OR corn OR cottonseed OR croton OR iodized OR ethiodized OR linseed OR palm OR "rice bran" OR safflower OR sesame OR soybean OR sunflower OR "tea tree") N3 oil*) ) OR KW ( ((plant OR eucalyptus OR rapeseed OR castor OR clove OR corn OR cottonseed OR croton OR iodized OR ethiodized OR linseed OR palm OR "rice bran" OR safflower OR sesame OR soybean OR sunflower OR "tea tree") N3 oil*) ) | 1,023 |
| S2 | TI ( ((herb* OR traditional* OR complementary OR alternative OR islam* OR folk OR indigenous* OR native* OR primitive OR arab* OR unani OR plant* OR garden* OR horticult* OR home) N3 (medicin* OR remedy OR remedies OR healing OR therap* OR pharmac*)) ) OR AB ( ((herb* OR traditional* OR complementary OR alternative OR islam* OR folk OR indigenous* OR native* OR primitive OR arab* OR unani OR plant* OR garden* OR horticult* OR home) N3 (medicin* OR remedy OR remedies OR healing OR therap* OR pharmac*)) ) OR KW ( ((herb* OR traditional* OR complementary OR alternative OR islam* OR folk OR indigenous* OR native* OR primitive OR arab* OR unani OR plant* OR garden* OR horticult* OR home) N3 (medicin* OR remedy OR remedies OR healing OR therap* OR pharmac*)) ) | 1,242 |
| S3 | TI ( (cam OR taim OR herbalism OR ethnobotan* OR ethnomedicin* OR phytotherap* OR aromatherap* OR ethnopharm* OR tea OR teas OR kombucha OR (plant* N3 extract*) OR (herb* N3 drug*)) ) OR AB ( (cam OR taim OR herbalism OR ethnobotan* OR ethnomedicin* OR phytotherap* OR aromatherap* OR ethnopharm* OR tea OR teas OR kombucha OR (plant* N3 extract*) OR (herb* N3 drug*)) ) OR KW ( (cam OR taim OR herbalism OR ethnobotan* OR ethnomedicin* OR phytotherap* OR aromatherap* OR ethnopharm* OR tea OR teas OR kombucha OR (plant* N3 extract*) OR (herb* N3 drug*)) ) | 1,180 |
| S4 | S1 OR S2 OR S3 | 3,124 |
| S5 | TI ( iraq* OR jordan* OR syria* OR palestin* OR lebanon OR lebanese OR gaza OR "west bank" OR ( east* N2 jerusalem ) ) OR AB ( iraq* OR jordan* OR syria* OR palestin* OR lebanon OR lebanese OR gaza OR "west bank" OR ( east* N2 jerusalem ) ) OR KW ( iraq* OR jordan* OR syria* OR palestin* OR lebanon OR lebanese OR gaza OR "west bank" OR ( east* N2 jerusalem ) ) OR GE ( iraq* OR jordan* OR syria* OR palestin* OR lebanon OR lebanese OR gaza OR "west bank" OR ( east* N2 jerusalem ) ) | 49,373 |
| S6 | S4 AND S5 | 218 |
| S7 | S6 AND Narrow by Language: - arabic  Narrow by Language: - english | 216 |
| **Total (with duplicates)** | | **216** |
| **Total (after removal of duplicates)** | | **142** |
|  | | |
|  | | |

| Database name: *CABI Digital Library*  Database platform: *CABI*  Date searched: *June 14, 2023*  Limits applied: *Languages (English, Arabic, Not specified)*  Alert set up: *Yes*  Note: *Kept 73 documents with language listed as not-specified* | | |
| --- | --- | --- |
| **Search** | **Query** | **Number of Results** |
| 1 | title:(((plant OR eucalyptus OR rapeseed OR castor OR clove OR corn OR cottonseed OR croton OR iodized OR ethiodized OR linseed OR palm OR "rice bran" OR safflower OR sesame OR soybean OR sunflower OR "tea tree") NEAR/3 oil*)) OR ab:(((plant OR eucalyptus OR rapeseed OR castor OR clove OR corn OR cottonseed OR croton OR iodized OR ethiodized OR linseed OR palm OR "rice bran" OR safflower OR sesame OR soybean OR sunflower OR "tea tree") NEAR/3 oil*)) OR subject:(((plant OR eucalyptus OR rapeseed OR castor OR clove OR corn OR cottonseed OR croton OR iodized OR ethiodized OR linseed OR palm OR "rice bran" OR safflower OR sesame OR soybean OR sunflower OR "tea tree") NEAR/3 oil*)) | 203,189 |
| 2 | title:(((herb* OR traditional* OR complementary OR alternative OR islam* OR folk OR indigenous* OR native* OR primitive OR arab* OR unani OR plant* OR garden* OR horticult* OR home) NEAR/3 (medicin* OR remedy OR remedies OR healing OR therap* OR pharmac*))) OR ab:(((herb* OR traditional* OR complementary OR alternative OR islam* OR folk OR indigenous* OR native* OR primitive OR arab* OR unani OR plant* OR garden* OR horticult* OR home) NEAR/3 (medicin* OR remedy OR remedies OR healing OR therap* OR pharmac*))) OR subject:(((herb* OR traditional* OR complementary OR alternative OR islam* OR folk OR indigenous* OR native* OR primitive OR arab* OR unani OR plant* OR garden* OR horticult* OR home) NEAR/3 (medicin* OR remedy OR remedies OR healing OR therap* OR pharmac*))) | 499,666 |
| 3 | title:((cam OR taim OR herbalism OR ethnobotan* OR ethnomedicin* OR phytotherap* OR aromatherap* OR ethnopharm* OR tea OR teas OR kombucha OR (plant* NEAR/3 extract*) OR (herb* NEAR/3 drug*))) OR ab:((cam OR taim OR herbalism OR ethnobotan* OR ethnomedicin* OR phytotherap* OR aromatherap* OR ethnopharm* OR tea OR teas OR kombucha OR (plant* NEAR/3 extract*) OR (herb* NEAR/3 drug*))) OR subject:((cam OR taim OR herbalism OR ethnobotan* OR ethnomedicin* OR phytotherap* OR aromatherap* OR ethnopharm* OR tea OR teas OR kombucha OR (plant* NEAR/3 extract*) OR (herb* NEAR/3 drug*))) | 660,691 |
| 4 | 1 OR 2 OR 3 | 933,920 |
| 5 | title:(iraq* OR jordan* OR syria* OR palestin* OR lebanon OR lebanese OR gaza OR "west bank" OR ( east* NEAR/2 jerusalem ) ) OR ab:(iraq* OR jordan* OR syria* OR palestin* OR lebanon OR lebanese OR gaza OR "west bank" OR ( east* NEAR/2 jerusalem ) ) OR subject:(iraq* OR jordan* OR syria* OR palestin* OR lebanon OR lebanese OR gaza OR "west bank" OR ( east* NEAR/2 jerusalem ) ) OR gl:(iraq* OR jordan* OR syria* OR palestin* OR lebanon OR lebanese OR gaza OR "west bank" OR ( east* NEAR/2 jerusalem ) ) | 51,970 |
| 6 | 4 AND 5 | 3,632 |
| 7 | 6 AND Refinements: Language = English OR Arabic OR not specified | 3,456 |
| **Total (with duplicates)** | | **3,456** |
| **Total (after removal of duplicates)** | | **2,206** |
|  | | |

| Database name: *Iraqi Academic Scientific Journals (IASJ)*  Database provider: *Ministry of Higher Education & Scientific Research of Iraq*  Date searched: *June 14-15, 2023*  Limits applied: *None*  Alert set up: *No* | |
| --- | --- |
| Search Strategy | |
| Using Advanced search, looked in the Abstract field for each of the below terms in combination with the country name variations [Iraq, Iraqi(s), Jordan, Jordanian(s), Lebanon, Lebanese, Palestine, Palestinian(s), Gaza, West Bank, East Jerusalem, Eastern Jerusalem, Syria, Syrian(s)]:  Complementary medicine, alternative medicine, unani, folk, indigenous plant(s), indigenous medicine, native plant(s), traditional medicine, traditional plant(s), Islamic plant(s), Islamic medicine, Islamic remedy, Islamic remedies, arab(ic) plant(s), arab(ic) medicine, arab(ic) remedy, arab(ic) remedies, herb(s), herbal, herbalism, medicinal plant(s), medicine plant(s), horticulture, horticultural, CAM, TAIM, ethnobotany, ethnobotanic, ethnobotanical, ethnobotanically, ethnomedicine, ethnomedicinal, ethnopharmacology, ethnopharmacologic, ethnopharmacological, phytotherapy, phytotherapies, phytotherapeutic, aromatherapy, aromatherapies, aromatherapeutic, ethnopharmacognosy, tea(s), kombucha | |
| **Total (with duplicates)** | **110** |
| **Total (after removal of duplicates)** | **92** |
|  | |

| Database name: *MEDLINE*  Database platform: *Ovid*  Date searched: *June 14, 2023*  Limits applied: *Language (English, Arabic)*  Alert set up: *Yes*  Note: *Ovid MEDLINE(R) and Epub Ahead of Print, In-Process, In-Data-Review & Other Non-Indexed Citations and Daily <1946 to June 13, 2023>*  Link to the search:  <https://ovidsp.ovid.com/ovidweb.cgi?T=JS&NEWS=N&PAGE=main&SHAREDSEARCHID=5HTnjiNtWXL68bakP9Rwkjyjk9rqEY3fr8a0fHxBAeeofMs7hPgTLzxclTV2mnh70> | | | |
| --- | --- | --- | --- |
| **Search** | **Query** | **Number of Results** | |
| 1 | Ethnopharmacology/ | 1,945 | |
| 2 | Plant Extracts/ | 137,612 | |
| 3 | exp Plant Oils/ | 42,310 | |
| 4 | exp Tea/ | 12,919 | |
| 5 | Teas, Herbal/ | 296 | |
| 6 | Herbal Medicine/ | 2,492 | |
| 7 | Plants, Medicinal/ | 62,487 | |
| 8 | Ethnobotany/ | 1,763 | |
| 9 | phytotherapy/ or aromatherapy/ | 42,052 | |
| 10 | Horticultural Therapy/ | 95 | |
| 11 | Complementary Therapies/ | 18,200 | |
| 12 | medicine, traditional/ or exp medicine, arabic/ | 13,011 | |
| 13 | ((plant or eucalyptus or rapeseed or castor or clove or corn or cottonseed or croton or iodized or ethiodized or linseed or palm or "rice bran" or safflower or sesame or soybean or sunflower or "tea tree") adj3 oil*).ti,ab,kw. | 38,123 | |
| 14 | ((herb* or traditional* or complementary or alternative or islam* or folk or indigenous* or native* or primitive or arab* or unani or plant* or garden* or horticult* or home) adj3 (medicin* or remedy or remedies or healing or therap* or pharmac*)).ti,ab,kw. | 184,041 | |
| 15 | (cam or taim or herbalism or ethnobotan* or ethnomedicin* or phytotherap* or aromatherap* or ethnopharm* or tea or teas or kombucha or (plant* adj3 extract*) or (herb* adj3 drug*)).ti,ab,kw. | 118,059 | |
| 16 | 1 or 2 or 3 or 4 or 5 or 6 or 7 or 8 or 9 or 10 or 11 or 12 or 13 or 14 or 15 | 492,805 | |
| 17 | iraq/ or jordan/ or lebanon/ or syria/ | 17,909 | |
| 18 | (Iraq* or Jordan* or syria* or palestin* or lebanon or lebanese or gaza or "west bank" or (east* adj2 jerusalem)).ti,ab,kw. | 45,511 | |
| 19 | 17 or 18 | 48,671 | |
| 20 | 16 and 19 | 1,216 | |
| 21 | limit 20 to (arabic or english) | 1,178 | |
| **Total (with duplicates)** | | **1,178** | |
| **Total (after removal of duplicates)** | | **103** | |
|  | | | |
|  | | | |
| Database name: *Scopus*  Database provider: *Elsevier*  Date searched: *June 14, 2023*  Limits applied: *Language (English, Arabic)*  Alert set up: *Yes* | | | |
| **Search** | **Query** | | **Number of Results** |
| 1 | TITLE-ABS-KEY ( ( ( plant OR eucalyptus OR rapeseed OR castor OR clove OR corn OR cottonseed OR croton OR iodized OR ethiodized OR linseed OR palm OR "rice bran" OR safflower OR sesame OR soybean OR sunflower OR "tea tree" ) W/3 oil* ) ) | | 161,764 |
| 2 | TITLE-ABS-KEY ( ( ( herb* OR traditional* OR complementary OR alternative OR islam* OR folk OR indigenous* OR native* OR primitive OR arab* OR unani OR plant* OR garden* OR horticult* OR home) W/3 ( medicin* OR remedy OR remedies OR healing OR therap* OR pharmac* ) ) ) | | 468,473 |
| 3 | TITLE-ABS-KEY ( ( cam OR taim OR herbalism OR ethnobotan* OR ethnomedicin* OR phytotherap* OR aromatherap* OR ethnopharm* OR tea OR teas OR kombucha OR ( plant* W/3 extract* ) OR ( herb* W/3 drug* ) ) ) | | 524,238 |
| 4 | 1 OR 2 OR 3 | | 986,865 |
| 5 | TITLE-ABS-KEY ( iraq* OR jordan* OR syria* OR palestin* OR lebanon OR lebanese OR gaza OR "west bank" OR ( east* W/2 jerusalem ) | | 189,440 |
| 6 | 4 AND 5 | | 3,105 |
| 7 | 6 AND ( LIMIT-TO ( LANGUAGE , "English" ) OR LIMIT-TO ( LANGUAGE , "Arabic" ) ) | | 2,972 |
| **Total (with duplicates)** | | | 2,972 |
| **Total (after removal of duplicates)** | | | **2,938** |
|  | | | |

| Database name: *Web of Science Core Collection*  Database provider: *Clarivate*  Date searched: *June 14, 2023*  Limits applied: *Language (English)*  Alert set up: *Yes*  Notes: *No documents in Arabic*  Link to the search strategy:  <https://www.webofscience.com/wos/woscc/summary/3e127b9c-e8fb-4faf-9697-1f6bd9d514ec-917ab3c6/relevance/1>  Collections searched: *Libraries’ subscribed products include Social Sciences Citation Index (SSCI) 1900 to 2023, Arts & Humanities Citation Index (AHCI) 1975 to 2023, Conference Proceedings Citation Index – Science (ISTP) 1990 to 2023, Emerging Sources Citation Index (ESCI) 2018 to 2023, Science Citation Index Expanded (SCI) 1900 to 2023, Conference Proceedings Citation Index – Social Sciences (ISSHP) 1990 to 2023* | | |
| --- | --- | --- |
| **Search** | **Query** | **Number of Results** |
| 1 | TS=(((plant OR eucalyptus OR rapeseed OR castor OR clove OR corn OR cottonseed OR croton OR iodized OR ethiodized OR linseed OR palm OR "rice bran" OR safflower OR sesame OR soybean OR sunflower OR "tea tree") NEAR/3 oil*)) | 111,373 |
| 2 | TS=(((herb* OR traditional* OR complementary OR alternative OR islam* OR folk OR indigenous* OR native* OR primitive OR arab* OR unani OR plant* OR garden* OR horticult* OR home) NEAR/3 (medicin* OR remedy OR remedies OR healing OR therap* OR pharmac*))) | 249,645 |
| 3 | TS=((cam OR taim OR herbalism OR ethnobotan* OR ethnomedicin* OR phytotherap* OR aromatherap* OR ethnopharm* OR tea OR teas OR kombucha OR (plant* NEAR/3 extract*) OR (herb* NEAR/3 drug*))) | 224,853 |
| 4 | #3 OR #2 OR #1 | 539,487 |
| 5 | TS=( iraq* OR jordan* OR syria* OR palestin* OR lebanon OR lebanese OR gaza OR "west bank" OR ( east* NEAR/2 jerusalem ) ) | 135,004 |
| 6 | #5 AND #4 | 1,470 |
| 7 | #6 and English (Languages) | 1,455 |
| **Total (with duplicates)** | | 1,455 |
| **Total (after removal of duplicates)** | | **271** |
|  | | |

1. Search engines:

| Search engine name: *Google Scholar (*[*https://scholar.google.com/*](https://scholar.google.com/)*)*  Search engine provider: *Google*  Date searched: *June 14-16, 2023*  Limits applied: *None*  Alert set up: *No* | |
| --- | --- |
| Search Strategy | |
| Using Advanced search, looked in the title field for each of the below terms in combination with the country name variations [Iraq, Iraqi(s), Jordan, Jordanian(s), Lebanon, Lebanese, Palestine, Palestinian(s), Gaza, West Bank, East Jerusalem, Eastern Jerusalem, Syria, Syrian(s)]:  Complementary medicine, alternative medicine, unani, folk, indigenous plant(s), indigenous medicine, native plant(s), traditional medicine, traditional plant(s), Islamic plant(s), Islamic medicine, Islamic remedy, Islamic remedies, arab(ic) plant(s), arab(ic) medicine, arab(ic) remedy, arab(ic) remedies, herb(s), herbal, herbalism, medicinal plant(s), medicine plant(s), horticulture, horticultural, CAM, TAIM, ethnobotany, ethnobotanic, ethnobotanical, ethnobotanically, ethnomedicine, ethnomedicinal, ethnopharmacology, ethnopharmacologic, ethnopharmacological, phytotherapy, phytotherapies, phytotherapeutic, aromatherapy, aromatherapies, aromatherapeutic, ethnopharmacognosy, tea(s), kombucha | |
| **Total (with duplicates)** | **680** |
| **Total (after removal of duplicates)** | **286** |
|  | |

##

## Additional Notes

| **1.** | **Indicate process for removing duplicates.** | Results were de-duplicated using EndNote X9.3.3 followed by manual double checking to ensure all duplicates are removed |
| --- | --- | --- |
| **2.** | **List filters or limits applied.** | Language = English, Arabic |
| **3.** | **Was this search based on a previous search such as a search used in a prior review?**  **(If yes, provide citation or source.)** | No |
| **4.** | **Was this search peer-reviewed? If so, by whom and what are their qualifications and area of**  **expertise?** | No |
| **5.** | **Other Notes** | N/A |
